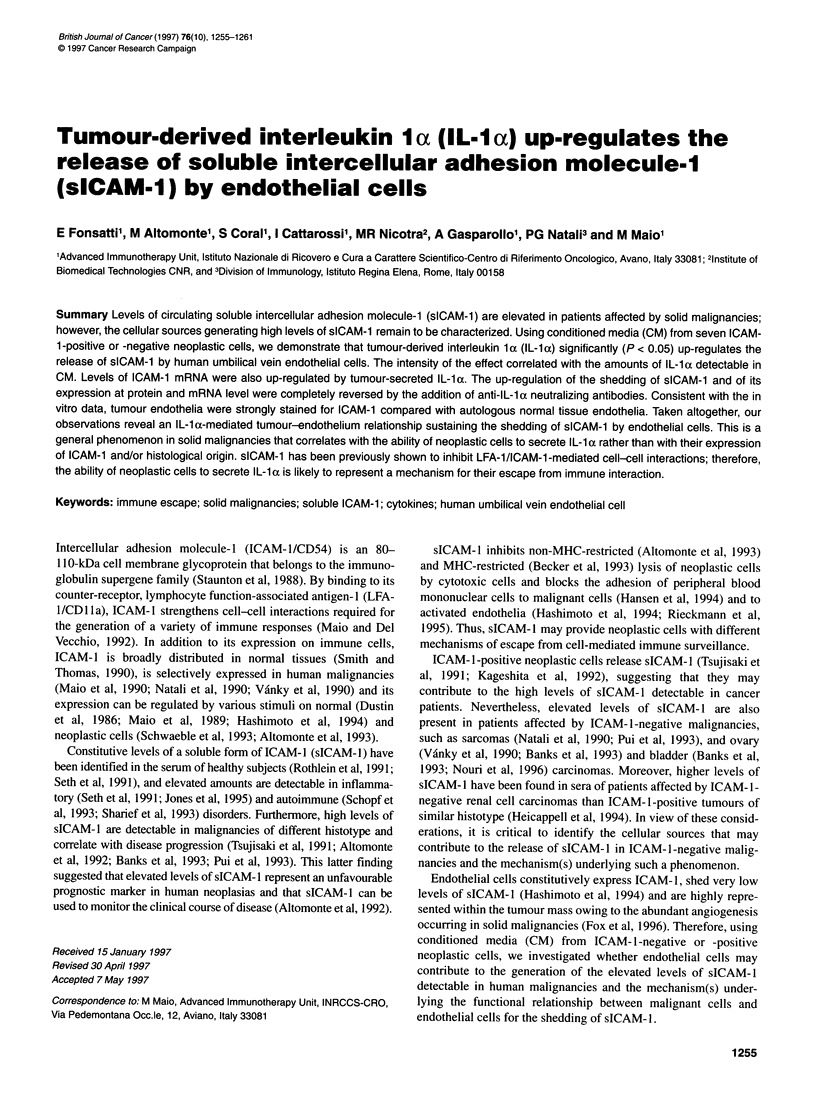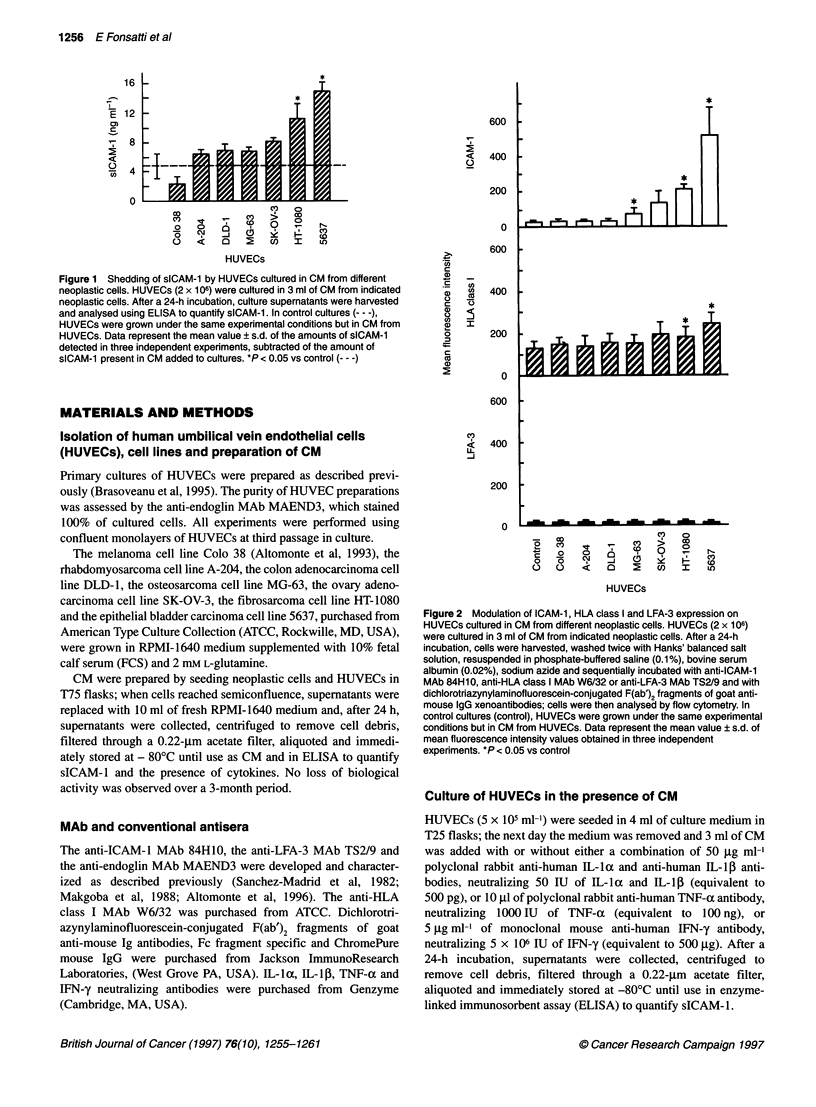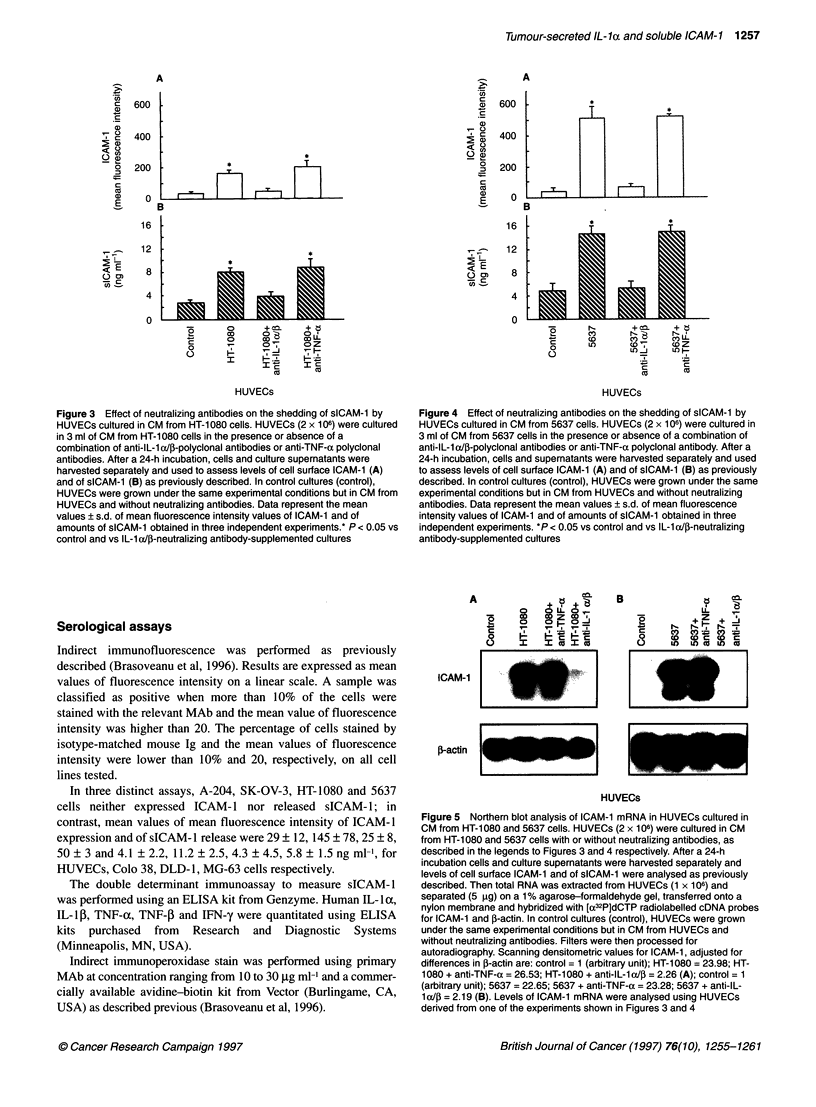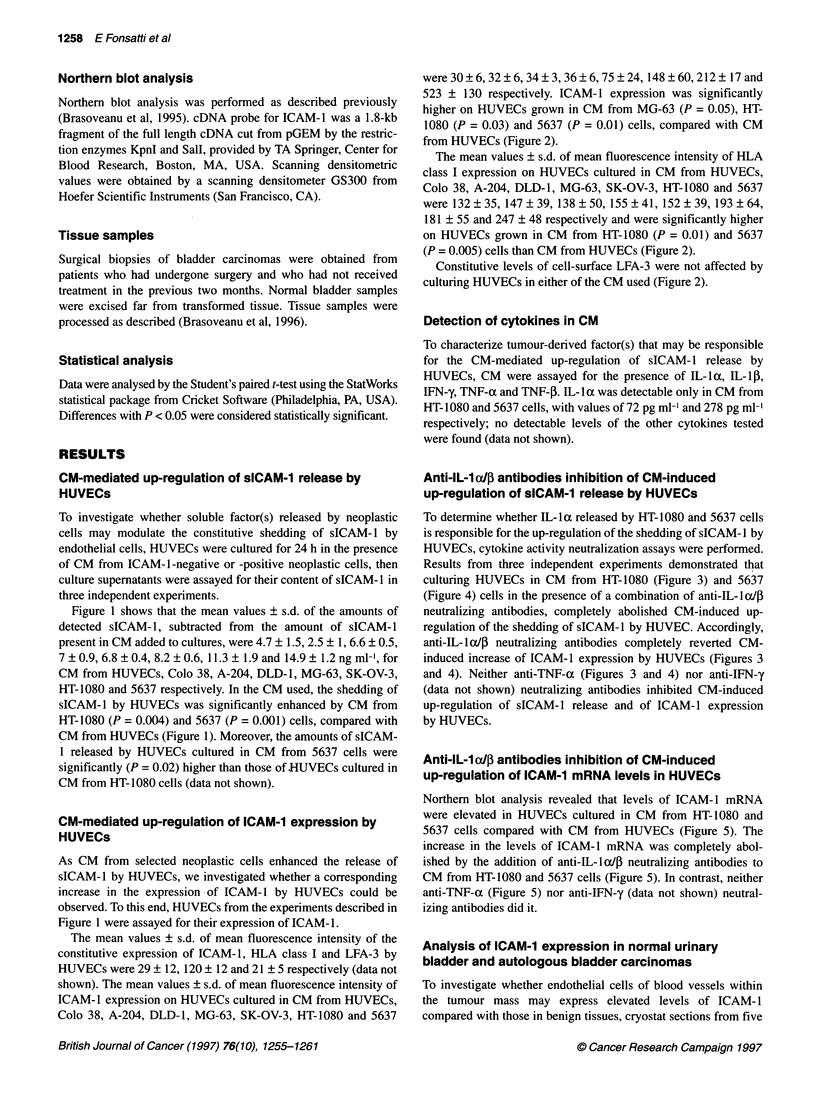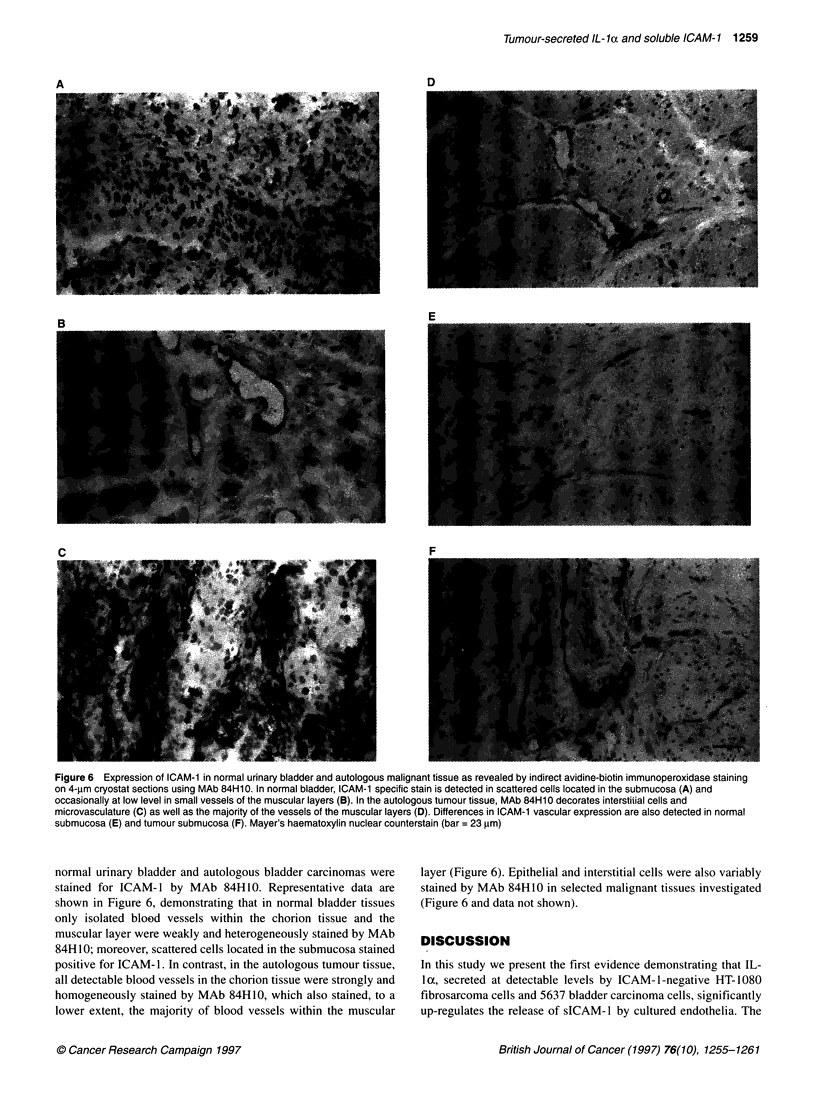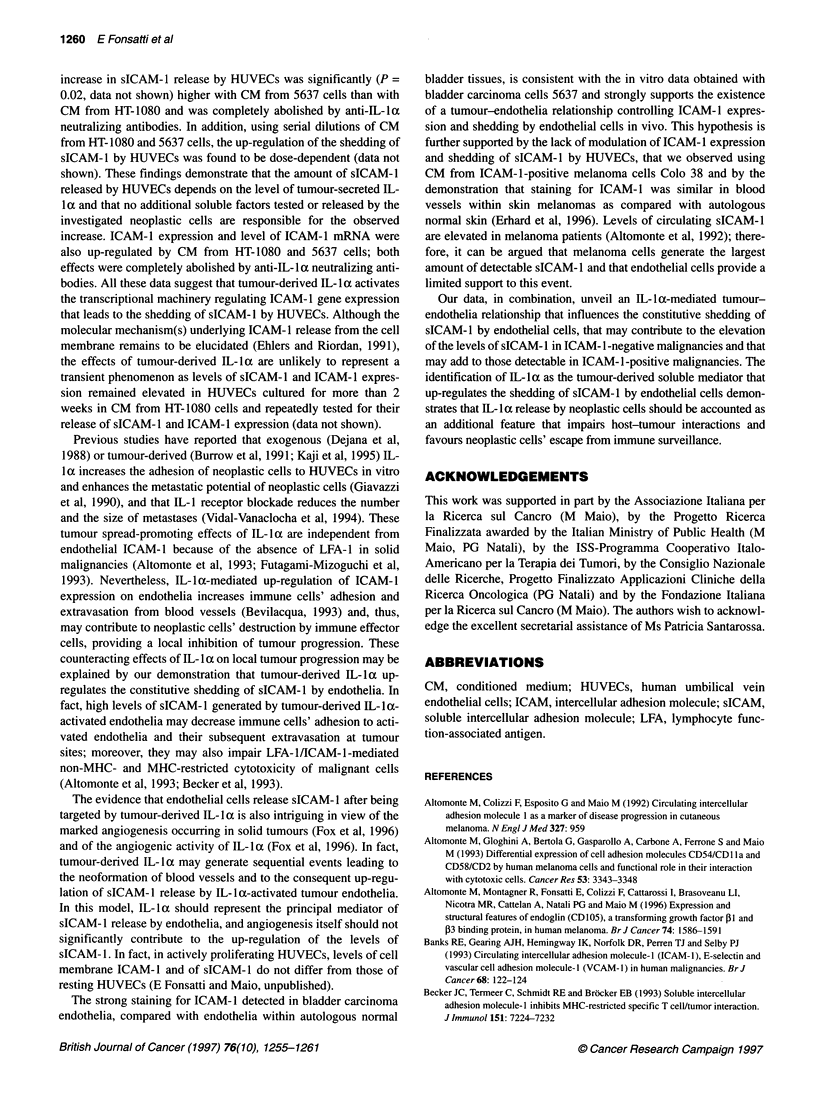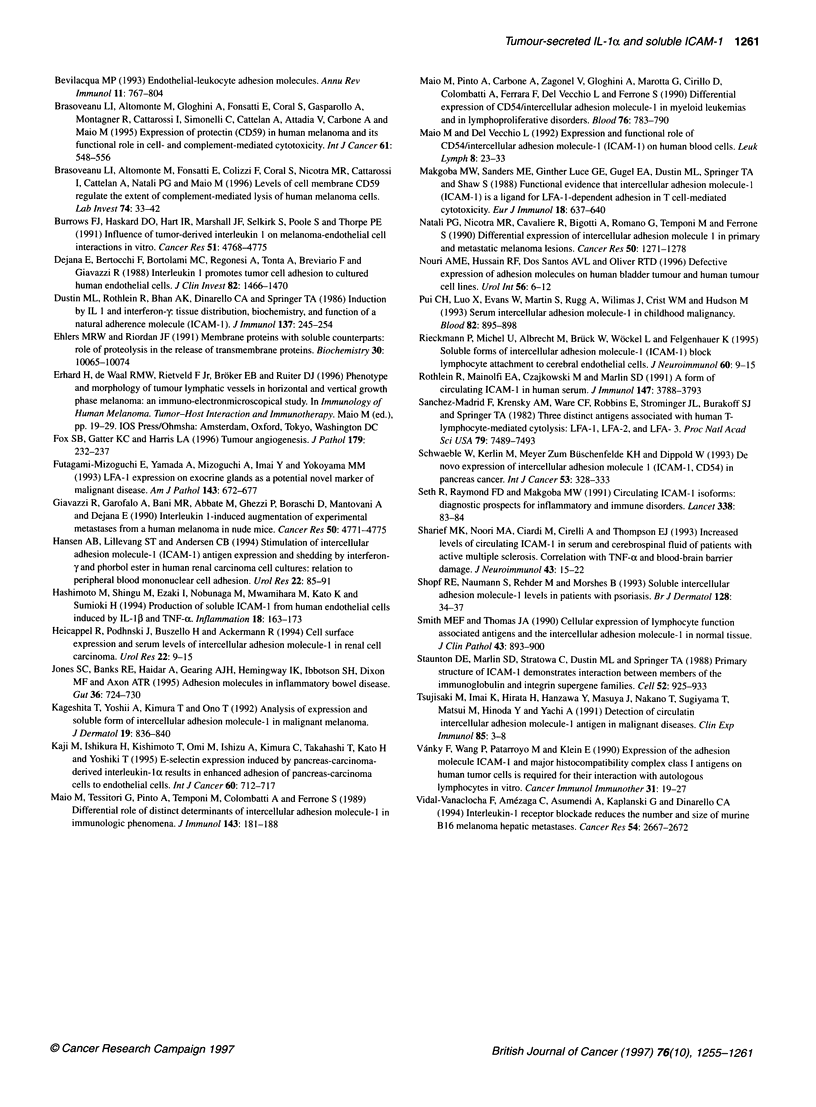# Tumour-derived interleukin 1alpha (IL-1alpha) up-regulates the release of soluble intercellular adhesion molecule-1 (sICAM-1) by endothelial cells.

**DOI:** 10.1038/bjc.1997.545

**Published:** 1997

**Authors:** E. Fonsatti, M. Altomonte, S. Coral, I. Cattarossi, M. R. Nicotra, A. Gasparollo, P. G. Natali, M. Maio

**Affiliations:** Advanced Immunotherapy Unit, Istituto Nazionale di Ricovero e Cura a Carattere Scientifico-Centro di Riferimento Oncologico, Avano, Italy.

## Abstract

**Images:**


British Journal of Cancer (1997) 76(10), 1255–1261
© 1997 Cancer Research Campaign

### Tumour-derived interleukin 1α (IL-1α) up-regulates the release of soluble intercellular adhesion molecule-1 (sICAM-1) by endothelial cells

**E Fonsatti[1], M Altomonte[1], S Coral[1], I Cattarossi[1], MR Nicotra[2], A Gasparollo[1], PG Natali[3] and M Maio[1]**

[1]Advanced Immunotherapy Unit, Istituto Nazionale di Ricovero e Cura a Carattere Scientifico-Centro di Riferimento Oncologico, Avano, Italy 33081; [2]Institute of Biomedical Technologies CNR, and [3]Division of Immunology, Istituto Regina Elena, Rome, Italy 00158

**Summary** Levels of circulating soluble intercellular adhesion molecule-1 (sICAM-1) are elevated in patients affected by solid malignancies; however, the cellular sources generating high levels of sICAM-1 remain to be characterized. Using conditioned media (CM) from seven ICAM-1-positive or -negative neoplastic cells, we demonstrate that tumour-derived interleukin 1α (IL-1α) significantly ($P < 0.05$) up-regulates the release of sICAM-1 by human umbilical vein endothelial cells. The intensity of the effect correlated with the amounts of IL-1α detectable in CM. Levels of ICAM-1 mRNA were also up-regulated by tumour-secreted IL-1α. The up-regulation of the shedding of sICAM-1 and of its expression at protein and mRNA level were completely reversed by the addition of anti-IL-1α neutralizing antibodies. Consistent with the in vitro data, tumour endothelia were strongly stained for ICAM-1 compared with autologous normal tissue endothelia. Taken altogether, our observations reveal an IL-1α-mediated tumour–endothelium relationship sustaining the shedding of sICAM-1 by endothelial cells. This is a general phenomenon in solid malignancies that correlates with the ability of neoplastic cells to secrete IL-1α rather than with their expression of ICAM-1 and/or histological origin. sICAM-1 has been previously shown to inhibit LFA-1/ICAM-1-mediated cell–cell interactions; therefore, the ability of neoplastic cells to secrete IL-1α is likely to represent a mechanism for their escape from immune interaction.

**Keywords:** immune escape; solid malignancies; soluble ICAM-1; cytokines; human umbilical vein endothelial cell

Intercellular adhesion molecule-1 (ICAM-1/CD54) is an 80–110-kDa cell membrane glycoprotein that belongs to the immunoglobulin supergene family (Staunton et al, 1988). By binding to its counter-receptor, lymphocyte function-associated antigen-1 (LFA-1/CD11a), ICAM-1 strengthens cell–cell interactions required for the generation of a variety of immune responses (Maio and Del Vecchio, 1992). In addition to its expression on immune cells, ICAM-1 is broadly distributed in normal tissues (Smith and Thomas, 1990), is selectively expressed in human malignancies (Maio et al, 1990; Natali et al, 1990; Vánky et al, 1990) and its expression can be regulated by various stimuli on normal (Dustin et al, 1986; Maio et al, 1989; Hashimoto et al, 1994) and neoplastic cells (Schwaeble et al, 1993; Altomonte et al, 1993).

Constitutive levels of a soluble form of ICAM-1 (sICAM-1) have been identified in the serum of healthy subjects (Rothlein et al, 1991; Seth et al, 1991), and elevated amounts are detectable in inflammatory (Seth et al, 1991; Jones et al, 1995) and autoimmune (Schopf et al, 1993; Sharief et al, 1993) disorders. Furthermore, high levels of sICAM-1 are detectable in malignancies of different histotype and correlate with disease progression (Tsujisaki et al, 1991; Altomonte et al, 1992; Banks et al, 1993; Pui et al, 1993). This latter finding suggested that elevated levels of sICAM-1 represent an unfavourable prognostic marker in human neoplasias and that sICAM-1 can be used to monitor the clinical course of disease (Altomonte et al, 1992).

*Received 15 January 1997*
*Revised 30 April 1997*
*Accepted 7 May 1997*

*Correspondence to:* M Maio, Advanced Immunotherapy Unit, INRCCS-CRO, Via Pedemontana Occ.le, 12, Aviano, Italy 33081

sICAM-1 inhibits non-MHC-restricted (Altomonte et al, 1993) and MHC-restricted (Becker et al, 1993) lysis of neoplastic cells by cytotoxic cells and blocks the adhesion of peripheral blood mononuclear cells to malignant cells (Hansen et al, 1994) and to activated endothelia (Hashimoto et al, 1994; Rieckmann et al, 1995). Thus, sICAM-1 may provide neoplastic cells with different mechanisms of escape from cell-mediated immune surveillance.

ICAM-1-positive neoplastic cells release sICAM-1 (Tsujisaki et al, 1991; Kageshita et al, 1992), suggesting that they may contribute to the high levels of sICAM-1 detectable in cancer patients. Nevertheless, elevated levels of sICAM-1 are also present in patients affected by ICAM-1-negative malignancies, such as sarcomas (Natali et al, 1990; Pui et al, 1993), and ovary (Vánky et al, 1990; Banks et al, 1993) and bladder (Banks et al, 1993; Nouri et al, 1996) carcinomas. Moreover, higher levels of sICAM-1 have been found in sera of patients affected by ICAM-1-negative renal cell carcinomas than ICAM-1-positive tumours of similar histotype (Heicappell et al, 1994). In view of these considerations, it is critical to identify the cellular sources that may contribute to the release of sICAM-1 in ICAM-1-negative malignancies and the mechanism(s) underlying such a phenomenon.

Endothelial cells constitutively express ICAM-1, shed very low levels of sICAM-1 (Hashimoto et al, 1994) and are highly represented within the tumour mass owing to the abundant angiogenesis occurring in solid malignancies (Fox et al, 1996). Therefore, using conditioned media (CM) from ICAM-1-negative or -positive neoplastic cells, we investigated whether endothelial cells may contribute to the generation of the elevated levels of sICAM-1 detectable in human malignancies and the mechanism(s) underlying the functional relationship between malignant cells and endothelial cells for the shedding of sICAM-1.

**Figure 1** Shedding of sICAM-1 by HUVECs cultured in CM from different neoplastic cells. HUVECs ($2 \times 10^6$) were cultured in 3 ml of CM from indicated neoplastic cells. After a 24-h incubation, culture supernatants were harvested and analysed using ELISA to quantify sICAM-1. In control cultures (- - -), HUVECs were grown under the same experimental conditions but in CM from HUVECs. Data represent the mean value ± s.d. of the amounts of sICAM-1 detected in three independent experiments, subtracted of the amount of sICAM-1 present in CM added to cultures. $^*P < 0.05$ vs control (- - -)

**Figure 2** Modulation of ICAM-1, HLA class I and LFA-3 expression on HUVECs cultured in CM from different neoplastic cells. HUVECs ($2 \times 10^6$) were cultured in 3 ml of CM from indicated neoplastic cells. After a 24-h incubation, cells were harvested, washed twice with Hanks' balanced salt solution, resuspended in phosphate-buffered saline (0.1%), bovine serum albumin (0.02%), sodium azide and sequentially incubated with anti-ICAM-1 MAb 84H10, anti-HLA class I MAb W6/32 or anti-LFA-3 MAb TS2/9 and with dichlorotriazynylaminofluorescein-conjugated F(ab')$_2$ fragments of goat anti-mouse IgG xenoantibodies; cells were then analysed by flow cytometry. In control cultures (control), HUVECs were grown under the same experimental conditions but in CM from HUVECs. Data represent the mean value ± s.d. of mean fluorescence intensity values obtained in three independent experiments. $^*P < 0.05$ vs control

### MATERIALS AND METHODS

### Isolation of human umbilical vein endothelial cells (HUVECs), cell lines and preparation of CM

Primary cultures of HUVECs were prepared as described previously (Brasoveanu et al, 1995). The purity of HUVEC preparations was assessed by the anti-endoglin MAb MAEND3, which stained 100% of cultured cells. All experiments were performed using confluent monolayers of HUVECs at third passage in culture.

The melanoma cell line Colo 38 (Altomonte et al, 1993), the rhabdomyosarcoma cell line A-204, the colon adenocarcinoma cell line DLD-1, the osteosarcoma cell line MG-63, the ovary adenocarcinoma cell line SK-OV-3, the fibrosarcoma cell line HT-1080 and the epithelial bladder carcinoma cell line 5637, purchased from American Type Culture Collection (ATCC, Rockwille, MD, USA), were grown in RPMI-1640 medium supplemented with 10% fetal calf serum (FCS) and 2 mM L-glutamine.

CM were prepared by seeding neoplastic cells and HUVECs in T75 flasks; when cells reached semiconfluence, supernatants were replaced with 10 ml of fresh RPMI-1640 medium and, after 24 h, supernatants were collected, centrifuged to remove cell debris, filtered through a 0.22-μm acetate filter, aliquoted and immediately stored at – 80°C until use as CM and in ELISA to quantify sICAM-1 and the presence of cytokines. No loss of biological activity was observed over a 3-month period.

### MAb and conventional antisera

The anti-ICAM-1 MAb 84H10, the anti-LFA-3 MAb TS2/9 and the anti-endoglin MAb MAEND3 were developed and characterized as described previously (Sanchez-Madrid et al, 1982; Makgoba et al, 1988; Altomonte et al, 1996). The anti-HLA class I MAb W6/32 was purchased from ATCC. Dichlorotriazynylaminofluorescein-conjugated F(ab')$_2$ fragments of goat anti-mouse Ig antibodies, Fc fragment specific and ChromePure mouse IgG were purchased from Jackson ImmunoResearch Laboratories, (West Grove PA, USA). IL-1α, IL-1β, TNF-α and IFN-γ neutralizing antibodies were purchased from Genzyme (Cambridge, MA, USA).

### Culture of HUVECs in the presence of CM

HUVECs ($5 \times 10^5$ ml$^{-1}$) were seeded in 4 ml of culture medium in T25 flasks; the next day the medium was removed and 3 ml of CM was added with or without either a combination of 50 μg ml$^{-1}$ polyclonal rabbit anti-human IL-1α and anti-human IL-1β antibodies, neutralizing 50 IU of IL-1α and IL-1β (equivalent to 500 pg), or 10 μl of polyclonal rabbit anti-human TNF-α antibody, neutralizing 1000 IU of TNF-α (equivalent to 100 ng), or 5 μg ml$^{-1}$ of monoclonal mouse anti-human IFN-γ antibody, neutralizing $5 \times 10^6$ IU of IFN-γ (equivalent to 500 μg). After a 24-h incubation, supernatants were collected, centrifuged to remove cell debris, filtered through a 0.22-μm acetate filter, aliquoted and immediately stored at –80°C until use in enzyme-linked immunosorbent assay (ELISA) to quantify sICAM-1.

© Cancer Research Campaign 1997

**Figure 3** Effect of neutralizing antibodies on the shedding of sICAM-1 by HUVECs cultured in CM from HT-1080 cells. HUVECs (2 x $10^6$) were cultured in 3 ml of CM from HT-1080 cells in the presence or absence of a combination of anti-IL-1α/β-polyclonal antibodies or anti-TNF-α polyclonal antibodies. After a 24-h incubation, cells and culture supernatants were harvested separately and used to assess levels of cell surface ICAM-1 (**A**) and of sICAM-1 (**B**) as previously described. In control cultures (control), HUVECs were grown under the same experimental conditions but in CM from HUVECs and without neutralizing antibodies. Data represent the mean values ± s.d. of mean fluorescence intensity values of ICAM-1 and of amounts of sICAM-1 obtained in three independent experiments.* $P < 0.05$ vs control and vs IL-1α/β-neutralizing antibody-supplemented cultures

**Figure 4** Effect of neutralizing antibodies on the shedding of sICAM-1 by HUVECs cultured in CM from 5637 cells. HUVECs (2 x $10^6$) were cultured in 3 ml of CM from 5637 cells in the presence or absence of a combination of anti-IL-1α/β-polyclonal antibodies or anti-TNF-α polyclonal antibody. After a 24-h incubation, cells and supernatants were harvested separately and used to assess levels of cell surface ICAM-1 (**A**) and of sICAM-1 (**B**) as previously described. In control cultures (control), HUVECs were grown under the same experimental conditions but in CM from HUVECs and without neutralizing antibodies. Data represent the mean values ± s.d. of mean fluorescence intensity values of ICAM-1 and of amounts of sICAM-1 obtained in three independent experiments. *$P < 0.05$ vs control and vs IL-1α/β-neutralizing antibody-supplemented cultures

### Serological assays

Indirect immunofluorescence was performed as previously described (Brasoveanu et al, 1996). Results are expressed as mean values of fluorescence intensity on a linear scale. A sample was classified as positive when more than 10% of the cells were stained with the relevant MAb and the mean value of fluorescence intensity was higher than 20. The percentage of cells stained by isotype-matched mouse Ig and the mean values of fluorescence intensity were lower than 10% and 20, respectively, on all cell lines tested.

In three distinct assays, A-204, SK-OV-3, HT-1080 and 5637 cells neither expressed ICAM-1 nor released sICAM-1; in contrast, mean values of mean fluorescence intensity of ICAM-1 expression and of sICAM-1 release were 29 ± 12, 145 ± 78, 25 ± 8, 50 ± 3 and 4.1 ± 2.2, 11.2 ± 2.5, 4.3 ± 4.5, 5.8 ± 1.5 ng ml$^{-1}$, for HUVECs, Colo 38, DLD-1, MG-63 cells respectively.

The double determinant immunoassay to measure sICAM-1 was performed using an ELISA kit from Genzyme. Human IL-1α, IL-1β, TNF-α, TNF-β and IFN-γ were quantitated using ELISA kits purchased from Research and Diagnostic Systems (Minneapolis, MN, USA).

Indirect immunoperoxidase stain was performed using primary MAb at concentration ranging from 10 to 30 μg ml$^{-1}$ and a commercially available avidine–biotin kit from Vector (Burlingame, CA, USA) as described previous (Brasoveanu et al, 1996).

**Figure 5** Northern blot analysis of ICAM-1 mRNA in HUVECs cultured in CM from HT-1080 and 5637 cells. HUVECs (2 x $10^6$) were cultured in CM from HT-1080 and 5637 cells with or without neutralizing antibodies, as described in the legends to Figures 3 and 4 respectively. After a 24-h incubation cells and culture supernatants were harvested separately and levels of cell surface ICAM-1 and of sICAM-1 were analysed as previously described. Then total RNA was extracted from HUVECs (1 x $10^6$) and separated (5 μg) on a 1% agarose–formaldehyde gel, transferred onto a nylon membrane and hybridized with [α$^{32}$P]dCTP radiolabelled cDNA probes for ICAM-1 and β-actin. In control cultures (control), HUVECs were grown under the same experimental conditions but in CM from HUVECs and without neutralizing antibodies. Filters were then processed for autoradiography. Scanning densitometric values for ICAM-1, adjusted for differences in β-actin are: control = 1 (arbitrary unit); HT-1080 = 23.98; HT-1080 + anti-TNF-α = 26.53; HT-1080 + anti-IL-1α/β = 2.26 (**A**); control = 1 (arbitrary unit); 5637 = 22.65; 5637 + anti-TNF-α = 23.28; 5637 + anti-IL-1α/β = 2.19 (**B**). Levels of ICAM-1 mRNA were analysed using HUVECs derived from one of the experiments shown in Figures 3 and 4

### Northern blot analysis

Northern blot analysis was performed as described previously (Brasoveanu et al, 1995). cDNA probe for ICAM-1 was a 1.8-kb fragment of the full length cDNA cut from pGEM by the restriction enzymes KpnI and SalI, provided by TA Springer, Center for Blood Research, Boston, MA, USA. Scanning densitometric values were obtained by a scanning densitometer GS300 from Hoefer Scientific Instruments (San Francisco, CA).

### Tissue samples

Surgical biopsies of bladder carcinomas were obtained from patients who had undergone surgery and who had not received treatment in the previous two months. Normal bladder samples were excised far from transformed tissue. Tissue samples were processed as described (Brasoveanu et al, 1996).

### Statistical analysis

Data were analysed by the Student's paired $t$-test using the StatWorks statistical package from Cricket Software (Philadelphia, PA, USA). Differences with $P < 0.05$ were considered statistically significant.

### RESULTS

### CM-mediated up-regulation of sICAM-1 release by HUVECs

To investigate whether soluble factor(s) released by neoplastic cells may modulate the constitutive shedding of sICAM-1 by endothelial cells, HUVECs were cultured for 24 h in the presence of CM from ICAM-1-negative or -positive neoplastic cells, then culture supernatants were assayed for their content of sICAM-1 in three independent experiments.

Figure 1 shows that the mean values ± s.d. of the amounts of detected sICAM-1, subtracted from the amount of sICAM-1 present in CM added to cultures, were 4.7 ± 1.5, 2.5 ± 1, 6.6 ± 0.5, 7 ± 0.9, 6.8 ± 0.4, 8.2 ± 0.6, 11.3 ± 1.9 and 14.9 ± 1.2 ng ml$^{-1}$, for CM from HUVECs, Colo 38, A-204, DLD-1, MG-63, SK-OV-3, HT-1080 and 5637 respectively. In the CM used, the shedding of sICAM-1 by HUVECs was significantly enhanced by CM from HT-1080 ($P = 0.004$) and 5637 ($P = 0.001$) cells, compared with CM from HUVECs (Figure 1). Moreover, the amounts of sICAM-1 released by HUVECs cultured in CM from 5637 cells were significantly ($P = 0.02$) higher than those of HUVECs cultured in CM from HT-1080 cells (data not shown).

### CM-mediated up-regulation of ICAM-1 expression by HUVECs

As CM from selected neoplastic cells enhanced the release of sICAM-1 by HUVECs, we investigated whether a corresponding increase in the expression of ICAM-1 by HUVECs could be observed. To this end, HUVECs from the experiments described in Figure 1 were assayed for their expression of ICAM-1.

The mean values ± s.d. of mean fluorescence intensity of the constitutive expression of ICAM-1, HLA class I and LFA-3 by HUVECs were 29 ± 12, 120 ± 12 and 21 ± 5 respectively (data not shown). The mean values ± s.d. of mean fluorescence intensity of ICAM-1 expression on HUVECs cultured in CM from HUVECs, Colo 38, A-204, DLD-1, MG-63, SK-OV-3, HT-1080 and 5637 were 30 ± 6, 32 ± 6, 34 ± 3, 36 ± 6, 75 ± 24, 148 ± 60, 212 ± 17 and 523 ± 130 respectively. ICAM-1 expression was significantly higher on HUVECs grown in CM from MG-63 ($P = 0.05$), HT-1080 ($P = 0.03$) and 5637 ($P = 0.01$) cells, compared with CM from HUVECs (Figure 2).

The mean values ± s.d. of mean fluorescence intensity of HLA class I expression on HUVECs cultured in CM from HUVECs, Colo 38, A-204, DLD-1, MG-63, SK-OV-3, HT-1080 and 5637 were 132 ± 35, 147 ± 39, 138 ± 50, 155 ± 41, 152 ± 39, 193 ± 64, 181 ± 55 and 247 ± 48 respectively and were significantly higher on HUVECs grown in CM from HT-1080 ($P = 0.01$) and 5637 ($P = 0.005$) cells than CM from HUVECs (Figure 2).

Constitutive levels of cell-surface LFA-3 were not affected by culturing HUVECs in either of the CM used (Figure 2).

### Detection of cytokines in CM

To characterize tumour-derived factor(s) that may be responsible for the CM-mediated up-regulation of sICAM-1 release by HUVECs, CM were assayed for the presence of IL-1α, IL-1β, IFN-γ, TNF-α and TNF-β. IL-1α was detectable only in CM from HT-1080 and 5637 cells, with values of 72 pg ml$^{-1}$ and 278 pg ml$^{-1}$ respectively; no detectable levels of the other cytokines tested were found (data not shown).

### Anti-IL-1α/β antibodies inhibition of CM-induced up-regulation of sICAM-1 release by HUVECs

To determine whether IL-1α released by HT-1080 and 5637 cells is responsible for the up-regulation of the shedding of sICAM-1 by HUVECs, cytokine activity neutralization assays were performed. Results from three independent experiments demonstrated that culturing HUVECs in CM from HT-1080 (Figure 3) and 5637 (Figure 4) cells in the presence of a combination of anti-IL-1α/β neutralizing antibodies, completely abolished CM-induced up-regulation of the shedding of sICAM-1 by HUVEC. Accordingly, anti-IL-1α/β neutralizing antibodies completely reverted CM-induced increase of ICAM-1 expression by HUVECs (Figures 3 and 4). Neither anti-TNF-α (Figures 3 and 4) nor anti-IFN-γ (data not shown) neutralizing antibodies inhibited CM-induced up-regulation of sICAM-1 release and of ICAM-1 expression by HUVECs.

### Anti-IL-1α/β antibodies inhibition of CM-induced up-regulation of ICAM-1 mRNA levels in HUVECs

Northern blot analysis revealed that levels of ICAM-1 mRNA were elevated in HUVECs cultured in CM from HT-1080 and 5637 cells compared with CM from HUVECs (Figure 5). The increase in the levels of ICAM-1 mRNA was completely abolished by the addition of anti-IL-1α/β neutralizing antibodies to CM from HT-1080 and 5637 cells (Figure 5). In contrast, neither anti-TNF-α (Figure 5) nor anti-IFN-γ (data not shown) neutralizing antibodies did it.

### Analysis of ICAM-1 expression in normal urinary bladder and autologous bladder carcinomas

To investigate whether endothelial cells of blood vessels within the tumour mass may express elevated levels of ICAM-1 compared with those in benign tissues, cryostat sections from five

 © Cancer Research Campaign 1997

**Figure 6** Expression of ICAM-1 in normal urinary bladder and autologous malignant tissue as revealed by indirect avidine-biotin immunoperoxidase staining on 4-μm cryostat sections using MAb 84H10. In normal bladder, ICAM-1 specific stain is detected in scattered cells located in the submucosa (**A**) and occasionally at low level in small vessels of the muscular layers (**B**). In the autologous tumour tissue, MAb 84H10 decorates interstitial cells and microvasculature (**C**) as well as the majority of the vessels of the muscular layers (**D**). Differences in ICAM-1 vascular expression are also detected in normal submucosa (**E**) and tumour submucosa (**F**). Mayer's haematoxylin nuclear counterstain (bar = 23 μm)

normal urinary bladder and autologous bladder carcinomas were stained for ICAM-1 by MAb 84H10. Representative data are shown in Figure 6, demonstrating that in normal bladder tissues only isolated blood vessels within the chorion tissue and the muscular layer were weakly and heterogeneously stained by MAb 84H10; moreover, scattered cells located in the submucosa stained positive for ICAM-1. In contrast, in the autologous tumour tissue, all detectable blood vessels in the chorion tissue were strongly and homogeneously stained by MAb 84H10, which also stained, to a lower extent, the majority of blood vessels within the muscular layer (Figure 6). Epithelial and interstitial cells were also variably stained by MAb 84H10 in selected malignant tissues investigated (Figure 6 and data not shown).

### DISCUSSION

In this study we present the first evidence demonstrating that IL-1α, secreted at detectable levels by ICAM-1-negative HT-1080 fibrosarcoma cells and 5637 bladder carcinoma cells, significantly up-regulates the release of sICAM-1 by cultured endothelia. The

© Cancer Research Campaign 1997

increase in sICAM-1 release by HUVECs was significantly ($P$ = 0.02, data not shown) higher with CM from 5637 cells than with CM from HT-1080 and was completely abolished by anti-IL-1α neutralizing antibodies. In addition, using serial dilutions of CM from HT-1080 and 5637 cells, the up-regulation of the shedding of sICAM-1 by HUVECs was found to be dose-dependent (data not shown). These findings demonstrate that the amount of sICAM-1 released by HUVECs depends on the level of tumour-secreted IL-1α and that no additional soluble factors tested or released by the investigated neoplastic cells are responsible for the observed increase. ICAM-1 expression and level of ICAM-1 mRNA were also up-regulated by CM from HT-1080 and 5637 cells; both effects were completely abolished by anti-IL-1α neutralizing antibodies. All these data suggest that tumour-derived IL-1α activates the transcriptional machinery regulating ICAM-1 gene expression that leads to the shedding of sICAM-1 by HUVECs. Although the molecular mechanism(s) underlying ICAM-1 release from the cell membrane remains to be elucidated (Ehlers and Riordan, 1991), the effects of tumour-derived IL-1α are unlikely to represent a transient phenomenon as levels of sICAM-1 and ICAM-1 expression remained elevated in HUVECs cultured for more than 2 weeks in CM from HT-1080 cells and repeatedly tested for their release of sICAM-1 and ICAM-1 expression (data not shown).

Previous studies have reported that exogenous (Dejana et al, 1988) or tumour-derived (Burrow et al, 1991; Kaji et al, 1995) IL-1α increases the adhesion of neoplastic cells to HUVECs in vitro and enhances the metastatic potential of neoplastic cells (Giavazzi et al, 1990), and that IL-1 receptor blockade reduces the number and the size of metastases (Vidal-Vanaclocha et al, 1994). These tumour spread-promoting effects of IL-1α are independent from endothelial ICAM-1 because of the absence of LFA-1 in solid malignancies (Altomonte et al, 1993; Futagami-Mizoguchi et al, 1993). Nevertheless, IL-1α-mediated up-regulation of ICAM-1 expression on endothelia increases immune cells' adhesion and extravasation from blood vessels (Bevilacqua, 1993) and, thus, may contribute to neoplastic cells' destruction by immune effector cells, providing a local inhibition of tumour progression. These counteracting effects of IL-1α on local tumour progression may be explained by our demonstration that tumour-derived IL-1α up-regulates the constitutive shedding of sICAM-1 by endothelia. In fact, high levels of sICAM-1 generated by tumour-derived IL-1α-activated endothelia may decrease immune cells' adhesion to activated endothelia and their subsequent extravasation at tumour sites; moreover, they may also impair LFA-1/ICAM-1-mediated non-MHC- and MHC-restricted cytotoxicity of malignant cells (Altomonte et al, 1993; Becker et al, 1993).

The evidence that endothelial cells release sICAM-1 after being targeted by tumour-derived IL-1α is also intriguing in view of the marked angiogenesis occurring in solid tumours (Fox et al, 1996) and of the angiogenic activity of IL-1α (Fox et al, 1996). In fact, tumour-derived IL-1α may generate sequential events leading to the neoformation of blood vessels and to the consequent up-regulation of sICAM-1 release by IL-1α-activated tumour endothelia. In this model, IL-1α should represent the principal mediator of sICAM-1 release by endothelia, and angiogenesis itself should not significantly contribute to the up-regulation of the levels of sICAM-1. In fact, in actively proliferating HUVECs, levels of cell membrane ICAM-1 and of sICAM-1 do not differ from those of resting HUVECs (E Fonsatti and Maio, unpublished).

The strong staining for ICAM-1 detected in bladder carcinoma endothelia, compared with endothelia within autologous normal bladder tissues, is consistent with the in vitro data obtained with bladder carcinoma cells 5637 and strongly supports the existence of a tumour–endothelia relationship controlling ICAM-1 expression and shedding by endothelial cells in vivo. This hypothesis is further supported by the lack of modulation of ICAM-1 expression and shedding of sICAM-1 by HUVECs, that we observed using CM from ICAM-1-positive melanoma cells Colo 38 and by the demonstration that staining for ICAM-1 was similar in blood vessels within skin melanomas as compared with autologous normal skin (Erhard et al, 1996). Levels of circulating sICAM-1 are elevated in melanoma patients (Altomonte et al, 1992); therefore, it can be argued that melanoma cells generate the largest amount of detectable sICAM-1 and that endothelial cells provide a limited support to this event.

Our data, in combination, unveil an IL-1α-mediated tumour–endothelia relationship that influences the constitutive shedding of sICAM-1 by endothelial cells, that may contribute to the elevation of the levels of sICAM-1 in ICAM-1-negative malignancies and that may add to those detectable in ICAM-1-positive malignancies. The identification of IL-1α as the tumour-derived soluble mediator that up-regulates the shedding of sICAM-1 by endothelial cells demonstrates that IL-1α release by neoplastic cells should be accounted as an additional feature that impairs host–tumour interactions and favours neoplastic cells' escape from immune surveillance.

### ACKNOWLEDGEMENTS

This work was supported in part by the Associazione Italiana per la Ricerca sul Cancro (M Maio), by the Progetto Ricerca Finalizzata awarded by the Italian Ministry of Public Health (M Maio, PG Natali), by the ISS-Programma Cooperativo Italo-Americano per la Terapia dei Tumori, by the Consiglio Nazionale delle Ricerche, Progetto Finalizzato Applicazioni Cliniche della Ricerca Oncologica (PG Natali) and by the Fondazione Italiana per la Ricerca sul Cancro (M Maio). The authors wish to acknowledge the excellent secretarial assistance of Ms Patricia Santarossa.

### ABBREVIATIONS

CM, conditioned medium; HUVECs, human umbilical vein endothelial cells; ICAM, intercellular adhesion molecule; sICAM, soluble intercellular adhesion molecule; LFA, lymphocyte function-associated antigen.

### REFERENCES

Altomonte M, Colizzi F, Esposito G and Maio M (1992) Circulating intercellular adhesion molecule 1 as a marker of disease progression in cutaneous melanoma. *N Engl J Med* **327**: 959

Altomonte M, Gloghini A, Bertola G, Gasparollo A, Carbone A, Ferrone S and Maio M (1993) Differential expression of cell adhesion molecules CD54/CD11a and CD58/CD2 by human melanoma cells and functional role in their interaction with cytotoxic cells. *Cancer Res* **53**: 3343–3348

Altomonte M, Montagner R, Fonsatti E, Colizzi F, Cattarossi I, Brasoveanu LI, Nicotra MR, Cattelan A, Natali PG and Maio M (1996) Expression and structural features of endoglin (CD105), a transforming growth factor β1 and β3 binding protein, in human melanoma. *Br J Cancer* **74**: 1586–1591

Banks RE, Gearing AJH, Hemingway IK, Norfolk DR, Perren TJ and Selby PJ (1993) Circulating intercellular adhesion molecule-1 (ICAM-1), E-selectin and vascular cell adhesion molecule-1 (VCAM-1) in human malignancies. *Br J Cancer* **68**: 122–124

Becker JC, Termeer C, Schmidt RE and Bröcker EB (1993) Soluble intercellular adhesion molecule-1 inhibits MHC-restricted specific T cell/tumor interaction. *J Immunol* **151**: 7224–7232

© Cancer Research Campaign 1997

Bevilacqua MP (1993) Endothelial-leukocyte adhesion molecules. *Annu Rev Immunol* **11**: 767–804

Brasoveanu LI, Altomonte M, Gloghini A, Fonsatti E, Coral S, Gasparollo A, Montagner R, Cattarossi I, Simonelli C, Cattelan A, Attadia V, Carbone A and Maio M (1995) Expression of protectin (CD59) in human melanoma and its functional role in cell- and complement-mediated cytotoxicity. *Int J Cancer* **61**: 548–556

Brasoveanu LI, Altomonte M, Fonsatti E, Colizzi F, Coral S, Nicotra MR, Cattarossi I, Cattelan A, Natali PG and Maio M (1996) Levels of cell membrane CD59 regulate the extent of complement-mediated lysis of human melanoma cells. *Lab Invest* **74**: 33–42

Burrows FJ, Haskard DO, Hart IR, Marshall JF, Selkirk S, Poole S and Thorpe PE (1991) Influence of tumor-derived interleukin 1 on melanoma-endothelial cell interactions in vitro. *Cancer Res* **51**: 4768–4775

Dejana E, Bertocchi F, Bortolami MC, Regonesi A, Tonta A, Breviario F and Giavazzi R (1988) Interleukin 1 promotes tumor cell adhesion to cultured human endothelial cells. *J Clin Invest* **82**: 1466–1470

Dustin ML, Rothlein R, Bhan AK, Dinarello CA and Springer TA (1986) Induction by IL 1 and interferon-γ: tissue distribution, biochemistry, and function of a natural adherence molecule (ICAM-1). *J Immunol* **137**: 245–254

Ehlers MRW and Riordan JF (1991) Membrane proteins with soluble counterparts: role of proteolysis in the release of transmembrane proteins. *Biochemistry* **30**: 10065–10074

Erhard H, de Waal RMW, Rietveld F Jr, Bröker EB and Ruiter DJ (1996) Phenotype and morphology of tumour lymphatic vessels in horizontal and vertical growth phase melanoma: an immuno-electronmicroscopical study. In *Immunology of Human Melanoma. Tumor–Host Interaction and Immunotherapy*. Maio M (ed.), pp. 19–29. IOS Press/Ohmsha: Amsterdam, Oxford, Tokyo, Washington DC

Fox SB, Gatter KC and Harris LA (1996) Tumour angiogenesis. *J Pathol* **179**: 232–237

Futagami-Mizoguchi E, Yamada A, Mizoguchi A, Imai Y and Yokoyama MM (1993) LFA-1 expression on exocrine glands as a potential novel marker of malignant disease. *Am J Pathol* **143**: 672–677

Giavazzi R, Garofalo A, Bani MR, Abbate M, Ghezzi P, Boraschi D, Mantovani A and Dejana E (1990) Interleukin 1-induced augmentation of experimental metastases from a human melanoma in nude mice. *Cancer Res* **50**: 4771–4775

Hansen AB, Lillevang ST and Andersen CB (1994) Stimulation of intercellular adhesion molecule-1 (ICAM-1) antigen expression and shedding by interferon-γ and phorbol ester in human renal carcinoma cell cultures: relation to peripheral blood mononuclear cell adhesion. *Urol Res* **22**: 85–91

Hashimoto M, Shingu M, Ezaki I, Nobunaga M, Mwamihara M, Kato K and Sumioki H (1994) Production of soluble ICAM-1 from human endothelial cells induced by IL-1β and TNF-α. *Inflammation* **18**: 163–173

Heicappel R, Podhnski J, Buszello H and Ackermann R (1994) Cell surface expression and serum levels of intercellular adhesion molecule-1 in renal cell carcinoma. *Urol Res* **22**: 9–15

Jones SC, Banks RE, Haidar A, Gearing AJH, Hemingway IK, Ibbotson SH, Dixon MF and Axon ATR (1995) Adhesion molecules in inflammatory bowel disease. *Gut* **36**: 724–730

Kageshita T, Yoshii A, Kimura T and Ono T (1992) Analysis of expression and soluble form of intercellular adhesion molecule-1 in malignant melanoma. *J Dermatol* **19**: 836–840

Kaji M, Ishikura H, Kishimoto T, Omi M, Ishizu A, Kimura C, Takahashi T, Kato H and Yoshiki T (1995) E-selectin expression induced by pancreas-carcinoma-derived interleukin-1α results in enhanced adhesion of pancreas-carcinoma cells to endothelial cells. *Int J Cancer* **60**: 712–717

Maio M, Tessitori G, Pinto A, Temponi M, Colombatti A and Ferrone S (1989) Differential role of distinct determinants of intercellular adhesion molecule-1 in immunologic phenomena. *J Immunol* **143**: 181–188

Maio M, Pinto A, Carbone A, Zagonel V, Gloghini A, Marotta G, Cirillo D, Colombatti A, Ferrara F, Del Vecchio L and Ferrone S (1990) Differential expression of CD54/intercellular adhesion molecule-1 in myeloid leukemias and in lymphoproliferative disorders. *Blood* **76**: 783–790

Maio M and Del Vecchio L (1992) Expression and functional role of CD54/intercellular adhesion molecule-1 (ICAM-1) on human blood cells. *Leuk Lymph* **8**: 23–33

Makgoba MW, Sanders ME, Ginther Luce GE, Gugel EA, Dustin ML, Springer TA and Shaw S (1988) Functional evidence that intercellular adhesion molecule-1 (ICAM-1) is a ligand for LFA-1-dependent adhesion in T cell-mediated cytotoxicity. *Eur J Immunol* **18**: 637–640

Natali PG, Nicotra MR, Cavaliere R, Bigotti A, Romano G, Temponi M and Ferrone S (1990) Differential expression of intercellular adhesion molecule 1 in primary and metastatic melanoma lesions. *Cancer Res* **50**: 1271–1278

Nouri AME, Hussain RF, Dos Santos AVL and Oliver RTD (1996) Defective expression of adhesion molecules on human bladder tumour and human tumour cell lines. *Urol Int* **56**: 6–12

Pui CH, Luo X, Evans W, Martin S, Rugg A, Wilimas J, Crist WM and Hudson M (1993) Serum intercellular adhesion molecule-1 in childhood malignancy. *Blood* **82**: 895–898

Rieckmann P, Michel U, Albrecht M, Brück W, Wöckel L and Felgenhauer K (1995) Soluble forms of intercellular adhesion molecule-1 (ICAM-1) block lymphocyte attachment to cerebral endothelial cells. *J Neuroimmunol* **60**: 9–15

Rothlein R, Mainolfi EA, Czajkowski M and Marlin SD (1991) A form of circulating ICAM-1 in human serum. *J Immunol* **147**: 3788–3793

Sanchez-Madrid F, Krensky AM, Ware CF, Robbins E, Strominger JL, Burakoff SJ and Springer TA (1982) Three distinct antigens associated with human T-lymphocyte-mediated cytolysis: LFA-1, LFA-2, and LFA- 3. *Proc Natl Acad Sci USA* **79**: 7489–7493

Schwaeble W, Kerlin M, Meyer Zum Büschenfelde KH and Dippold W (1993) De novo expression of intercellular adhesion molecule 1 (ICAM-1, CD54) in pancreas cancer. *Int J Cancer* **53**: 328–333

Seth R, Raymond FD and Makgoba MW (1991) Circulating ICAM-1 isoforms: diagnostic prospects for inflammatory and immune disorders. *Lancet* **338**: 83–84

Sharief MK, Noori MA, Ciardi M, Cirelli A and Thompson EJ (1993) Increased levels of circulating ICAM-1 in serum and cerebrospinal fluid of patients with active multiple sclerosis. Correlation with TNF-α and blood-brain barrier damage. *J Neuroimmunol* **43**: 15–22

Shopf RE, Naumann S, Rehder M and Morshes B (1993) Soluble intercellular adhesion molecule-1 levels in patients with psoriasis. *Br J Dermatol* **128**: 34–37

Smith MEF and Thomas JA (1990) Cellular expression of lymphocyte function associated antigens and the intercellular adhesion molecule-1 in normal tissue. *J Clin Pathol* **43**: 893–900

Staunton DE, Marlin SD, Stratowa C, Dustin ML and Springer TA (1988) Primary structure of ICAM-1 demonstrates interaction between members of the immunoglobulin and integrin supergene families. *Cell* **52**: 925–933

Tsujisaki M, Imai K, Hirata H, Hanzawa Y, Masuya J, Nakano T, Sugiyama T, Matsui M, Hinoda Y and Yachi A (1991) Detection of circulatin intercellular adhesion molecule-1 antigen in malignant diseases. *Clin Exp Immunol* **85**: 3–8

Vánky F, Wang P, Patarroyo M and Klein E (1990) Expression of the adhesion molecule ICAM-1 and major histocompatibility complex class I antigens on human tumor cells is required for their interaction with autologous lymphocytes in vitro. *Cancer Immunol Immunother* **31**: 19–27

Vidal-Vanaclocha F, Amézaga C, Asumendi A, Kaplanski G and Dinarello CA (1994) Interleukin-1 receptor blockade reduces the number and size of murine B16 melanoma hepatic metastases. *Cancer Res* **54**: 2667–2672

© Cancer Research Campaign 1997